# *Selaginella tamariscina* extract suppresses TPA-induced invasion and metastasis through inhibition of MMP-9 in human nasopharyngeal carcinoma HONE-1 cells

**DOI:** 10.1186/1472-6882-13-234

**Published:** 2013-09-23

**Authors:** Chung-Han Hsin, Buor-Chang Wu, Chun-Yi Chuang, Shun-Fa Yang, Yi-Hsien Hsieh, Hsin-Yu Ho, Huang-Pin Lin, Mu-Kuan Chen, Chiao-Wen Lin

**Affiliations:** 1 School of Medicine, Chung Shan Medical University, Taichung, Taiwan; 2 Department of Otolaryngology, Chung Shan Medical University Hospital, Taichung, Taiwan; 3 School of Dentistry, Chung Shan Medical University, Taichung, Taiwan; 4 Department of Dentistry, Chung Shan Medical University Hospital, Taichung, Taiwan; 5 Institute of Medicine, Chung Shan Medical University, Taichung, Taiwan; 6 Department of Medical Research, Chung Shan Medical University Hospital, Taichung, Taiwan; 7 Department of Biochemistry, School of Medicine, Chung Shan Medical University, Taichung, Taiwan; 8 Department of Otorhinolaryngology-Head and Neck Surgery, Changhua Christian Hospital, Changhua, Taiwan; 9 Institute of Oral Sciences, Chung Shan Medical University, 110 Chien-Kuo N. Road, Section 1, Taichung, Taiwan

**Keywords:** *Selaginella tamariscina*, MMP-9, Invasion, Nasopharyngeal carcinoma

## Abstract

**Background:**

Nasopharyngeal carcinoma (NPC) is known for its high incidence of neck lymph node metastasis, which represents poor prognosis. The present study aimed to examine the anti-metastatic properties of *Selaginella tamariscina* extract (STE) in human nasopharyngeal carcinoma HONE-1 cells *in vitro*.

**Methods:**

Cell viability was examined by MTT assay, whereas cell motility was measured by invasive, migration and would healing assays. Real-time PCR, and promoter assays confirmed the inhibitory effects of STE on matrix metalloproteinase-9 (MMP-9) mRNA level in HONE-1 cells.

**Results:**

The STE inhibits 12-O-tetradecanoylphorbol-13-acetate (TPA)-induced HONE-1 cell migration and invasion in a concentration-dependent manner. By zymographic and Western blot analyses, STE was shown to inhibit the activities and expression of MMP-9. Treatment of STE on TPA-induced HONE-1 cells inhibited MMP-9 expression and ERK1/2 phosphorylation without affecting JNK and p38 phosphorylation.

**Conclusions:**

STE inhibits MMP-9 expression and HONE-1 cell metastasis. Its inhibitory effects may involve the Src/FAK/ERK 1/2 pathway. STE may have the potential of being an anti-metastatic agent against NPC.

## Background

Nasopharyngeal carcinoma (NPC) remains endemic among ethnic Chinese and the Inuits of Alaska. It is a distinct entity of head and neck cancers because of its characteristic epidemiology, pathogenesis, and association with the Epstein-Barr virus [1]. Metastasis of cancer cells to the neck lymph nodes, which can occur in up to 75% of NPC patients, represents an adverse prognostic factor of the disease [2]. Distant metastases, such as those to the lungs, liver, and bone, remain a major cause of treatment failure [3]. Metastasis is a phenomenon composed of multiple sequential cascades and various cyto-physiologic changes, including reduction of tumor cell adhesion, degradation of extracellular matrix (ECM), enhancement of cell motility, and promotion of neo-vascularization [4]. Thus, a degradation of the ECM and components of the basement membrane caused by the concerted action of proteinases like matrix metalloproteinases (MMPs), cathepsins, and plasminogen activators (PA) play an important role in tumor invasion and metastasis [5,6]. Among these enzymes, MMP-2 and MMP-9 can degrade most ECM components and are profoundly involved in the development of cancer invasion and metastasis [7,8]. Therefore, the inhibition of MMP-2 or MMP-9-mediated migration or invasion may be a preventive method of limiting cancer metastasis.

*Selaginella tamariscina* (Beauv.) is a traditional Chinese herbal medicine for chronic trachitis. Its major constituents are flavonoids (e.g. amentoflavone, hinokiflavone, sotetsuflavone, and apogenin) and saccharides (e.g. trehalose, d-glucose, dfructose and d-rhamnose) [9-11]. Previous studies have demonstrated that *Selaginella tamariscina* possesses anti-bacterial, anti-hypertensive, and anti-hyperglycemic effects [10,12,13]. Moreover, *Selaginella tamariscina* has been shown to have anti-tumor activities, probably via an expression of the p53 tumor suppressor gene and an induction of G1 arrest in the cell cycle against certain tumor cell lines [14]. Recently, Yang et al. found that *Selaginella tamariscina* extract (STE) can down-regulate the expression of MMPs and u-PA, and inhibit the invasion and metastatic activities of lung cancer cells [15]. There is, however, no data about the anti-metastatic potential of STE on NPC cancer cells. Thus, this study examined the effects of aqueous extracts of *Selaginella tamariscina* with potential anti-metastatic properties in 12-O-tetradecanoylphorbol-13-acetate (TPA)-treated human NPC HONE-1 cells *in vitro* to investigate the signaling pathway of the process.

## Methods

### Preparation of *Selaginella tamariscina* extracts

*Selaginella tamariscina* (Beauv.) leaves were purchased from herb stores in Taichung, Taiwan and the *Selaginella tamariscina* extracts (STE) were prepared as described previously [16]. The plant material was identified at the Department of Biochemistry of Chung Shan Medical University in Taichung and a voucher specimen is deposited. Briefly, 100 g of air-dried leaves were boiled at 70°C for 24 hours with 500 mL of 50% ethanol. The extraction procedure was repeated twice. The solvent was removed from the combined extract using a vacuum rotary evaporator. The filtrate was then lyophilized and stored at −20°C until further studies were to be conducted. A voucher specimen was deposited in the National Research Institute of Chinese Medicine, Taiwan [16].

The extraction yield was 2.8% (w/w) and the chemical profile of STE was analyzed using high-pressure liquid chromatograms (HPLC)-mass spectrometer. Briefly, the STE was analyzed by Hitachi L-6200 with an L-4500 Diode Array detector with a PE Sciex Qstar Pulsar ESI-TOF mass spectrometer. Samples (10 μl) were injected onto a Merck LiChrospher 100 RP-18 column (4mm×250 mm). The column was equilibrated in 0.05% acetic acid/water (solution A) and elution of the components was achieved by increasing the concentration of solution B (100% acetonitrile) from 0 to 100% in 30 min at a flow rate of 1 ml/min. Absorbance was monitored at 254 nm. The molecular masses of the peaks were determined from electro-spray ionization mass spectra using a multiply-charged ion profile based on the modified method of Chang et al. [17]. For subsequent experiments, the STE powder was dissolved in dimethyl sulfate (DMSO) to achieve designed concentrations (0, 25, 50, 75, and 100 μg/mL).

### Cell and cell culture

A human nasopharyngeal carcinoma cell line from ATCC (Manassas, VA), HONE-1 cells, was cultured in RPMI-1640 medium (Life Technologies, Grand Island, NY), 10% fetal bovine serum, 2 mM glutamine, 100 U/ml penicillin, and 100 μg/ml streptomycin. All cell cultures were maintained at 37°C in a humidified atmosphere of 5% CO2. For STE treatment, appropriate amounts of stock solution of STE were added into the culture medium to achieve the indicated concentrations. The cells were then incubated for the indicated time periods. Dimethyl sulfoxide solution without STE was used as blank reagent.

### Analysis of cell viability (MTT assay)

To evaluate the cytotoxicity of STE, an MTT colorimetric assay was performed to determine cell viability [18]. Cells were seeded in 24-well plates at a density of 1×10^5^ cells per well and treated with 0, 25, 50, 75, 100, 150 and 200 μg/mL of STE at 37°C in 5% CO_2_ for 24 h and 48 h. At the end of the exposure period, the cells were washed with PBS and incubated with 0.8 mL of MTT (Sigma chemical Co., St. Louis, MO, USA) per well (final concentration, 0.5 mg/mL) at 37°C in 5% CO_2_ for 4 h. The viable cell number was directly proportional to the production of formazan following solubilization with isopropanol, which was measured spectrophotometrically at 563 nm (Beckman Spectrophotometer DU640, Beckman Instruments, Fullerton, CA, USA).

### Cell migration and invasion assays

Cell migration and invasion were assayed according to the methods described by Chu et al. [19]. After treatment with STE for 24 h, the surviving HONE-1 cells were harvested and seeded to a Boyden chamber (Neuro Probe, Cabin John, MD, USA) at 10^4^ cells per well in serum-free medium, and then incubated for 24 h at 37°C. To determine cell migration, the cells were seeded into the Boyden chamber on membrane filters that were not coated with Matrigel. The filters were then air-dried for 5 h in a laminar flow hood. The migrating cells were fixed with methanol and stained with Giemsa. The cell numbers were counted by light microscopy.

For the invasion assay, 10 μL Matrigel (25 mg/50 mL; BD Biosciences, MA, USA) was applied to 8 μm pore size polycarbonate membrane filters. The bottom chamber contained standard medium. The invasion of cells treated or untreated with STE was measured as in the migration assay.

### Determination of MMP-9 activity by zymography

The activities of MMP-9 in the conditional medium were measured by gelatin zymography protease assays as previously described [20]. Briefly, collected media of an appropriate volume were prepared with SDS sample buffer without boiling or reduction, and subjected to 0.1% gelatin-8% SDS-PAGE electrophoresis. After electrophoresis, the gels were washed with 2.5% Triton X-100 and incubated in a reaction buffer (40 mM Tris–HCl, pH 8.0; 10mM CaCl2 and 0.01% NaN3) at 37°C for 12 h. The gel was stained with Coomassie brilliant blue R-250 for visualization.

### RNA preparation and TaqMan quantitative real-time PCR

Total RNA was isolated from cancer cells using Trizol (Life Technologies, Grand Island, NY) according to the manufacturer’s instructions. Quantitative real-time PCR analysis was performed using TaqMan one-step PCR Master Mix (Applied Biosystems). Total cDNA (100 ng) was added per 25 μl reaction with MMP-9 or GAPDH primers and TaqMan probes. The MMP-9 and GAPDH primers and probes were designed using commercial software (ABI PRISM Sequence Detection System; Applied Biosystems). The oligonucleotide sequences of TaqMan probes and primers were described in Table 1. Quantitative real-time PCR assays were conducted in triplicate on a StepOnePlus sequence detection system. Threshold was set above the non-template control background and within the linear phase of target gene amplification to calculate the cycle number at which the transcript was detected [21].

**Table 1:** Primers list for real-time PCR assay

**Primers used in real-time PCR**	**Sequence (5’ to 3’)**
MMP-9 (Hs00957562_m1)	(FAM)- GGCGCTCATGTACCCTATGT
GAPDH ( Hs99999905_m1)	(FAM)-GGCGCCTGGTCACCAGGGCTGCTTT

### Transfection and MMP-9 promoter-driven luciferase assays

The HONE-1 cells were seeded at a concentration of 5 x10^4^ cells per well in 6-well cell culture plates. After 24 h of incubation, pGL3-basic (vector) and MMP-9 promoter plasmid were co-transfected with a β-galactosidase expression vector (pCH110) into cells using Turbofect (Fermentas, Carlsbad, CA) as previously described [20]. After 12 h of transfection, the cells were treated with vehicle or STE (0~50 μg/mL) for 24 h. The cell lysates were harvested and luciferase activity was determined using a luciferase assay kit. The value of the luciferase activity was normalized to transfection efficiency and monitored by β-galactosidase expression.

### Western blot analysis for determining molecular pathway

Total cell lysates or nuclear extracts were prepared as previously described [22]. The cell lysates were separated in a 10% polyacrylamide gel and transferred onto a nitrocellulose membrane. The blot was subsequently incubated with 5% non-fat milk in Tris-buffered saline (20 mM Tris, 137 mM NaCl, pH 7.6) for 1 h to block non-specific binding, and then overnight with polyclonal antibodies against three MAPKs (ERK 1/2, JNK ½, and p38), Src, FAK, and β-actin with the specific antibodies for unphosphorylated or phosphorylated forms. The blots were then incubated with horseradish peroxidase goat anti-rabbit or anti-mouse IgG for 1 h.

Signal was detected by using an enhanced chemi-luminescence (ECL) commercial kit (Amersham Biosciences). The relative photographic density was quantitated by scanning the photographic negatives on a gel documentation and analysis system (AlphaImager 2000, Alpha Innotech Corporation, San Leandro, CA, USA).

### Statistical analysis

Statistically significant differences were calculated using the Student’s t-test (Sigma-Stat 2.0, Jandel Scientific, San Rafael, CA, USA). Significance was set at *p*<0.05. The values are the means ± standard deviation (SD) of at least three independent experiments.

## Results

### Effect of STE on the viability of HONE-1 cells

**Figure 1:**
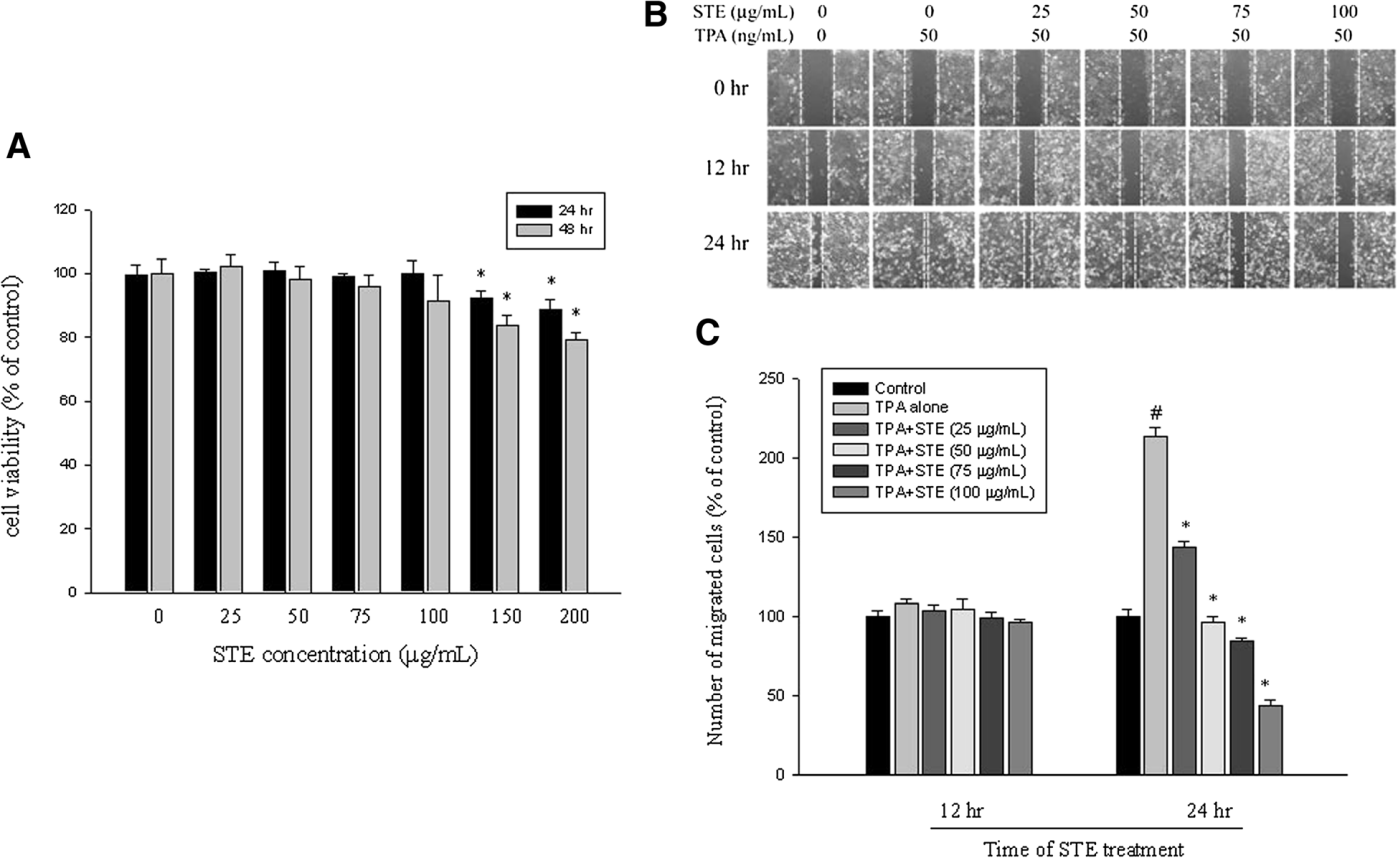
**Effect of STE on cell viability and *in vitro *wound closure in HONE-1 cell. (A)** HONE-1 cells were treated with STE (0, 25, 50, 75, 100, 150, and 200 μg/mL) for 24 h and 48 h before a MTT assay for cell viability. The values are means±SD of at least three independent experiments. **(B)** HONE-1 cells were wounded and then treated with vehicle (DMSO) or STE (0, 25, 50, 75 and 100 μg/mL) in the presence or absence of TPA (50 ng/mL) for 0, 12, and 24 h in 0.5% FBS-containing medium. In the different time points, phase-contrast pictures of the wounds at three different locations were taken. **(C)** Cells migrating into the wound area were counted using the dashed line as time zero. A quantitative assessment of the mean number of cells in the denuded zone was the mean±SD (n=3). *#p<*0.05 compared to the vehicle group; *p<0.05 compared with the TPA treatment group.

The effects of STE on the viability of HONE-1 cells in 24 h and 48 h were assessed by MTT assay and the cytotoxic effects of various STE concentrations (0, 25, 50, 75, 100, 150, and 200 μg/mL) were shown in Figure 1A. The MTT assay showed that at the highest concentration (200 μg/mL), STE altered HONE-1 cell viability. As such, a lower concentration range of STE (0, 25, 50, 75, and 100 μg/mL) was used for all subsequent experiments.

### Inhibitory effects of STE on In vitro wound closure, migration and invasion of HONE-1 cells

**Figure 2:**
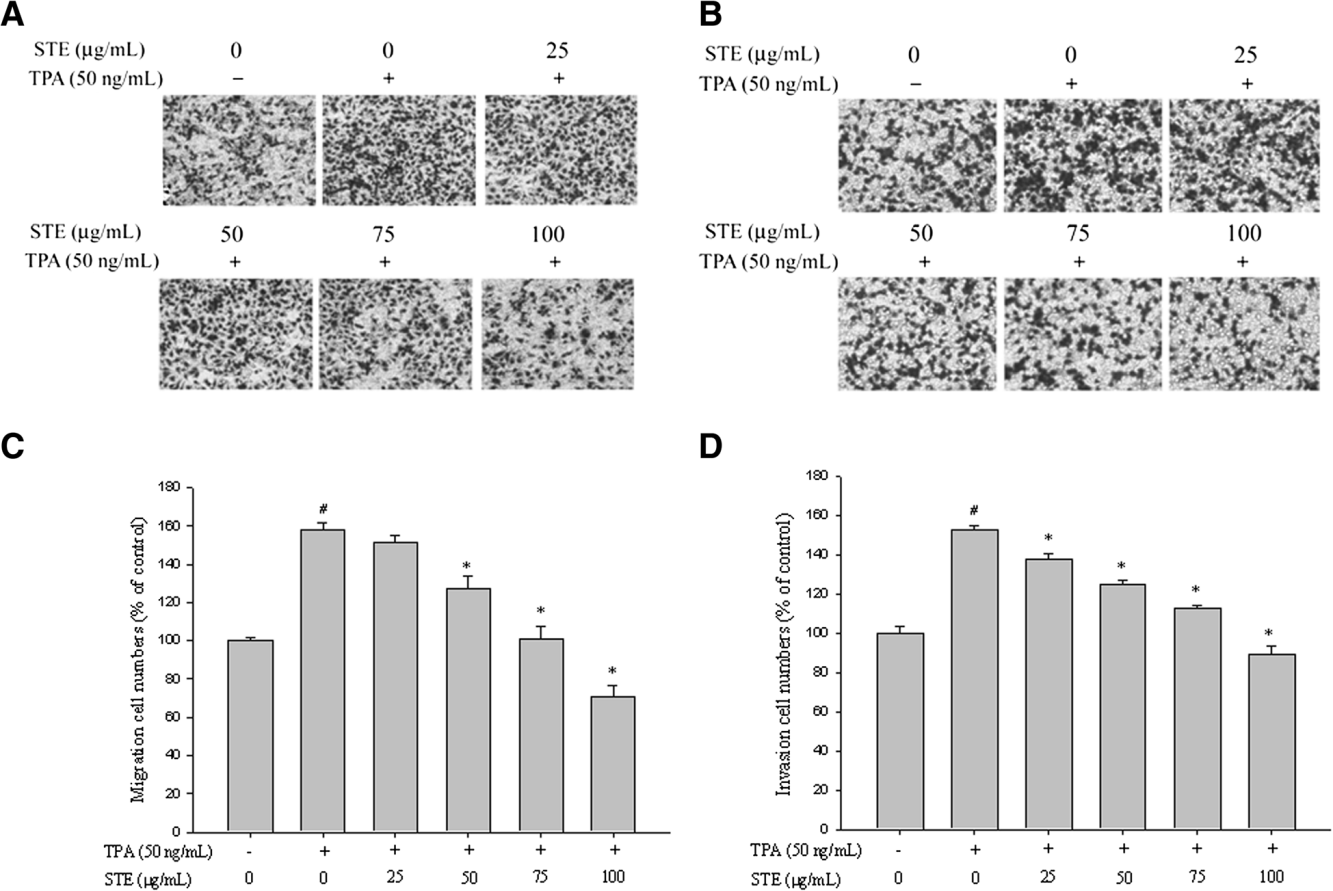
**Effects of STE on cell migration and invasion in HONE-1 cells. (A)** Cell migration and **(B)** invasion were measured using a Boyden chamber for 16 and 24 h with polycarbonate filters, respectively. The **(C)** migration and **(D)** invasion abilities of HONE-1 cells were quantified by counting the number of cells that invaded the underside of the porous polycarbonate, as described in the *Materials and Methods* section. The values represent the means±SD of at least three independent experiments. *#p<*0.05 compared with the vehicle group; *p<0.05 compared to the TPA treatment group.

Findings from a wound closure assay determined the effects of STE on the migration of HONE-1 cells and contained representative photographs of HONE-1 cells migrating into the scratch wounds during STE treatment (Figure 1B). In the wound closure assay, STE significantly reduced the motility of HONE-1 cells at 24 h (*p*<0.05) (Figure 2C).

The migration and invasion assays with the Boyden chamber were used to investigate effects of STE on HONE-1 cells. In the well where TPA alone was applied, there was a 58±3.8% increase in cell migration ability compared to the control well (*p*<0.001). Significant reductions (*p*<0.05) on the migration of HONE-1 cells were observed when STE concentrations were beyond 25 μg /mL (Figure 2A). In the well where TPA alone was applied, there was a 52.5±2.3% increase in cell invasion ability compared to the control well (*p*<0.001). There were significant reductions (*p*<0.05) on the invasion of HONE-1 cells in all STE concentrations (Figure 2B).

### Effects of STE on the TPA-induced MMP-9 activity in HONE-1 cells

**Figure 3:**
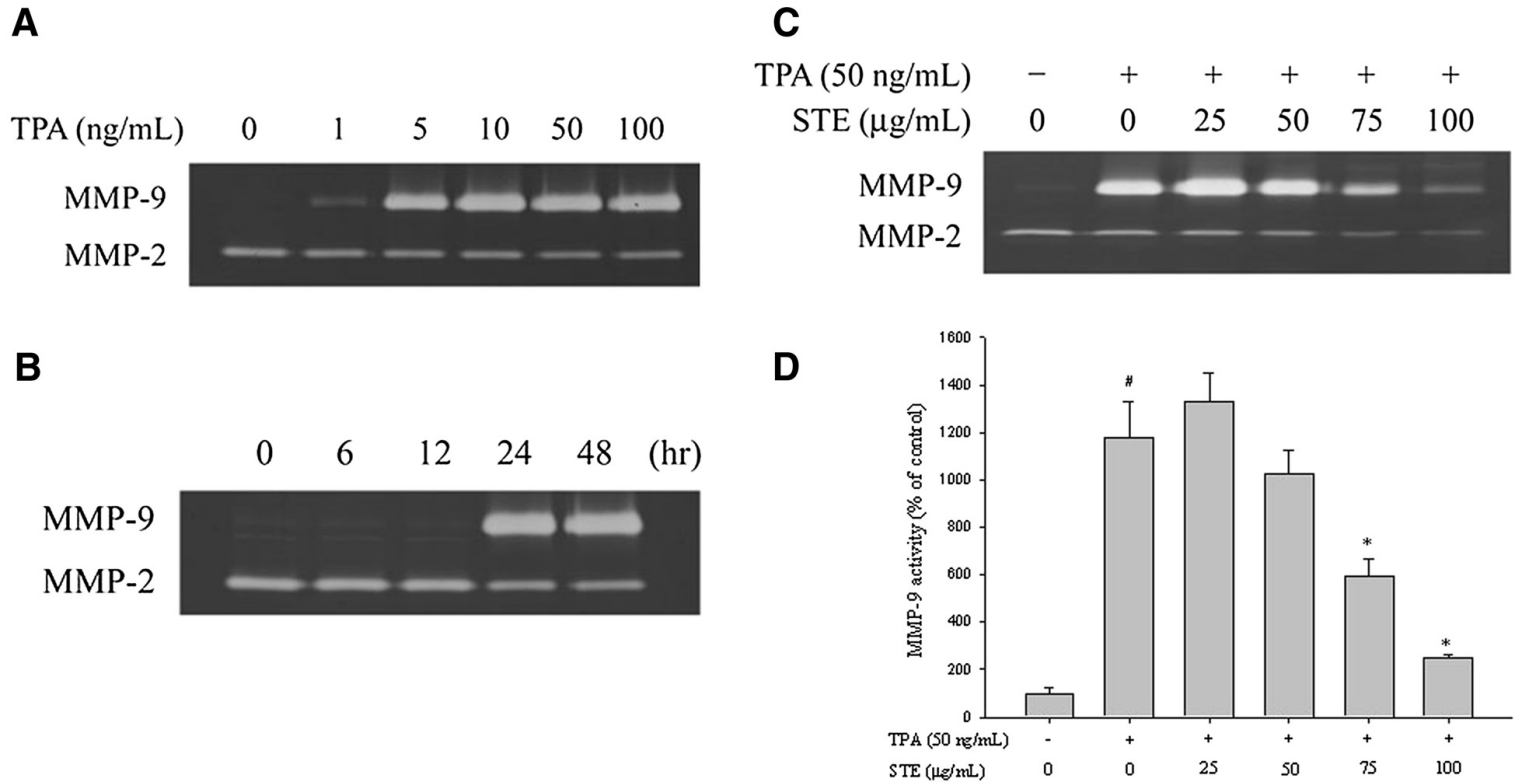
**Effects of TPA and STE on MMP-9 activity. (A)** HONE-1 cells were treated with TPA (1, 5, 10, 50, and 100 ng/mL) for 24 h. **(B)** HONE-1 cells were treated with TPA (50 ng/mL) for 6, 12, 24 and 48 h. **(C**-**D)** HONE-1 cells were treated with STE (0, 25, 50, 75, and 100 μg/mL) in the presence or absence of TPA (50 ng/mL) for 24 h. All of the cells were subjected to gelatin zymography analysis to determine the activities of MMP-2 and MMP-9. The values represented the means±SD of at least three independent experiments. *#p<*0.05 compared to the vehicle group; *p<0.05 compared to the TPA treatment group.

Gelatin zymography assay was used to investigate the enzymatic activity of MMP-2 and MMP-9 on HONE-1 cells following TPA treatment. In the gelatin zymography assay, TPA significantly increased the MMP-9 activity of HONE-1 cells in a dose- and time-dependent manner (*p*<0.05) (Figure 3A and 3B) while the MMP-2 activity remained changed. The assay revealed that enzymatic activity of MMP-9 could reach up to 1177.7±154.1% of the control sample after TPA induction (Figure 3C). Except at low concentrations (25 μg/mL), STE could reduce MMP-9 activity inn HONE-1 cells in a concentration-dependent manner (Figure 3C and 3D).

### Effects of STE on the mRNA expression and promoter activity of TPA-induced MMP-9 in HONE-1 cells

**Figure 4:**
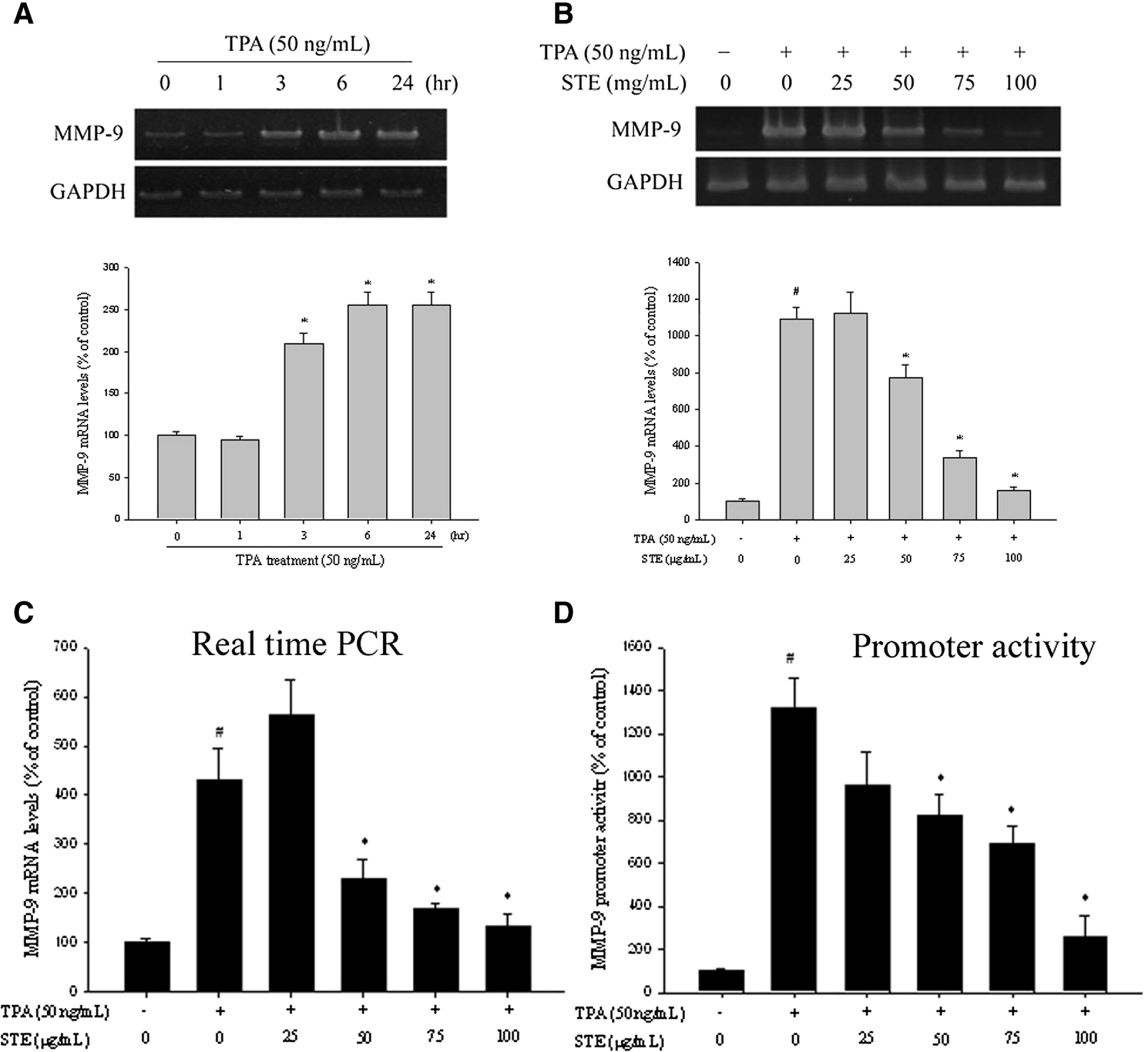
**Effects of STE on mRNA level and MMP-9 promoter activity. (A)** HONE-1 cells were treated with TPA (50 ng/mL) for 1, 3, 6, and 24 h and then subjected to RT-PCR to analyze the mRNA levels of MMP-9. **(B**-**C)** HONE-1 cells were treated with STE (0, 25, 50, 75, and 100 μg/mL) in the presence or absence of TPA (50 ng/mL) for 6 h and then subjected to **(B)** RT-PCR and **(C)** TaqMan quantitative real-time PCR to analyze the mRNA levels of MMP-9. **(D)** HONE-1 cells were treated with STE (0, 25, 50, 75, and 100 μg/mL) in the presence or absence of TPA (50 ng/mL) for 6 h and then subjected to promoter-driven luciferase assays to analyze the promoter activity of MMP-9. The values represented the means±SD of at least three independent experiments. *#p<*0.05 compared to the vehicle group; *p<0.05 compared to the TPA treatment group.

In the RT-PCR assay, TPA significantly increased the MMP-9 activity of HONE-1 cells in a time-dependent manner (*p*<0.05) (Figure 4A). The inhibitory effects of various STE concentrations on the mRNA expression of MMP-9 were investigated using RT-PCR at 6 h after TPA treatment. Except at low concentrations (25 μg /mL), STE significantly reduced (*p*<0.05) MMP-9 mRNA expression of HONE-1 cells in a concentration-dependent manner (Figure 4B).

To further assess the effects of various STE concentrations on the mRNA expression of MMP-9 in HONE-1 cells, real-time PCR with GAPDH as internal control was utilized. The treatment of TPA boosted the expression of MMP-9 mRNA to 428.6±65.4%, while STE significantly reduced (*p*<0.05) the TPA-induced MMP-9 mRNA expression except at the concentrations of 25 μg/mL (Figure 4C). The luciferase activities of MMP-9 were also significantly suppressed (Figure 4D). These results suggested that STE regulated MMP-9 expression, at least partially, at the transcriptional level.

### Effects of STE on focal adhesion kinase (FAK) activation

**Figure 5:**
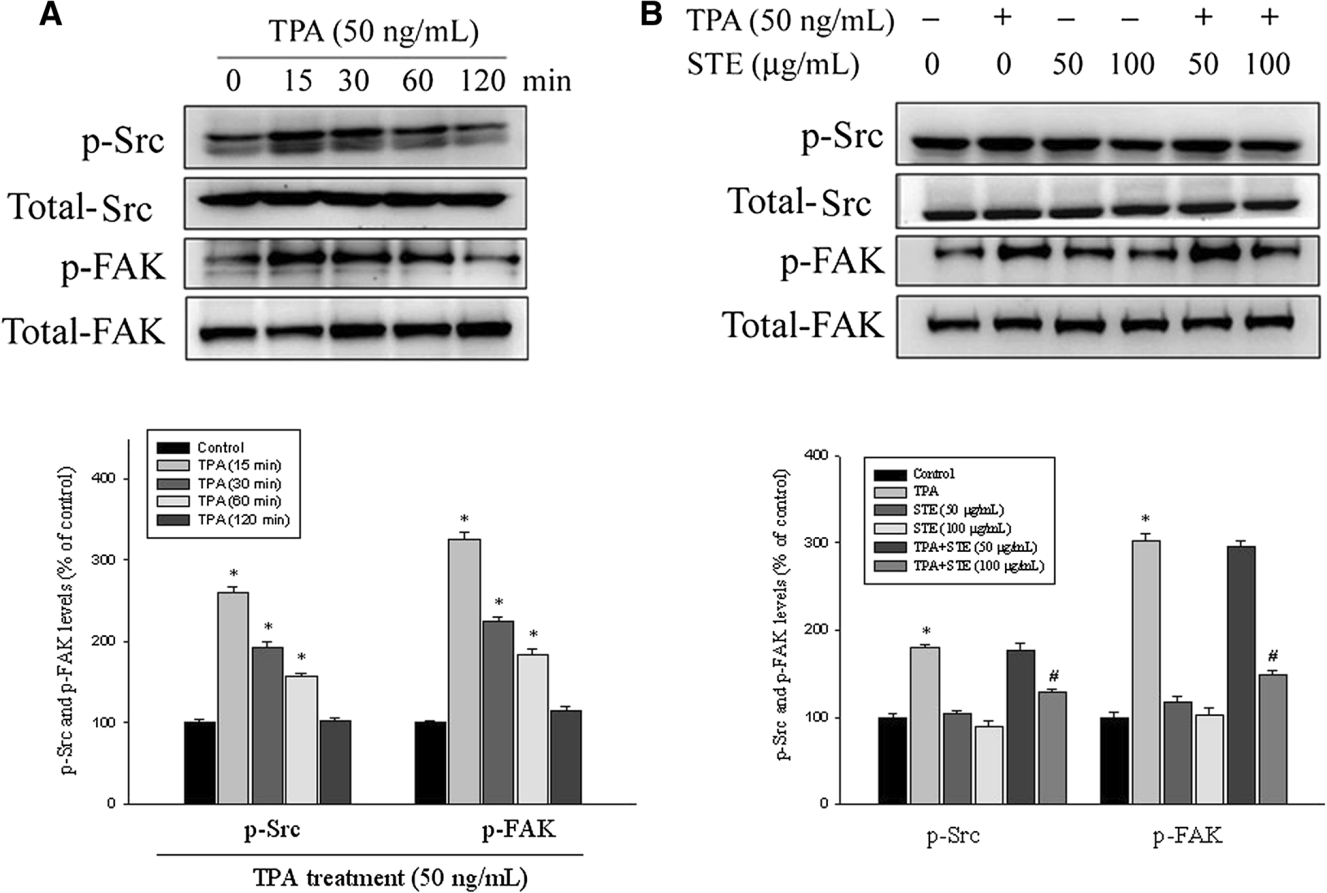
**Effects of TPA and STE on the phosphorylation level of Src and FAK. (A)** HONE-1 cells were treated with TPA (50 ng/mL) for 15, 30, 60, and 120 min and then subjected to Western blotting to analyze Src and FAK levels. **(B)** HONE-1 cells were treated with STE (0, 50, and 100 μg/mL) in the presence or absence of TPA (50 ng/mL) for 6 h and then subjected to Western blotting to to analyze Src and FAK levels. Quantitative results of phosphorylation levels of Src or FAK were adjusted with total Src and FAK protein level. The values represented the means±SD of at least three independent experiments. *p<0.05 compared to the vehicle group; *#p<*0.05 compared to the TPA treatment group.

The investigation on the molecular regulation of cell migration indicated the involvement of Src and FAK in STE treatment. Changes in FAK activation in these cells were further evaluated using antibodies directed against the FAK phosphorylation site Tyr925. Western blotting showed that TPA significantly increased the Src and FAK phosphorylation of HONE-1 cells in a time-dependent manner (*p*<0.05) (Figure 5A). Moreover, STE reduced the phosphorylation of Src and FAK in HONE-1 cells (Figure 5B) (*p*<0.05), suggesting that STE inhibited HONE-1 cell migration, at least in part, through the regulation of Src and FAK phosphorylation.

### Effects of STE on MAPK pathways

**Figure 6:**
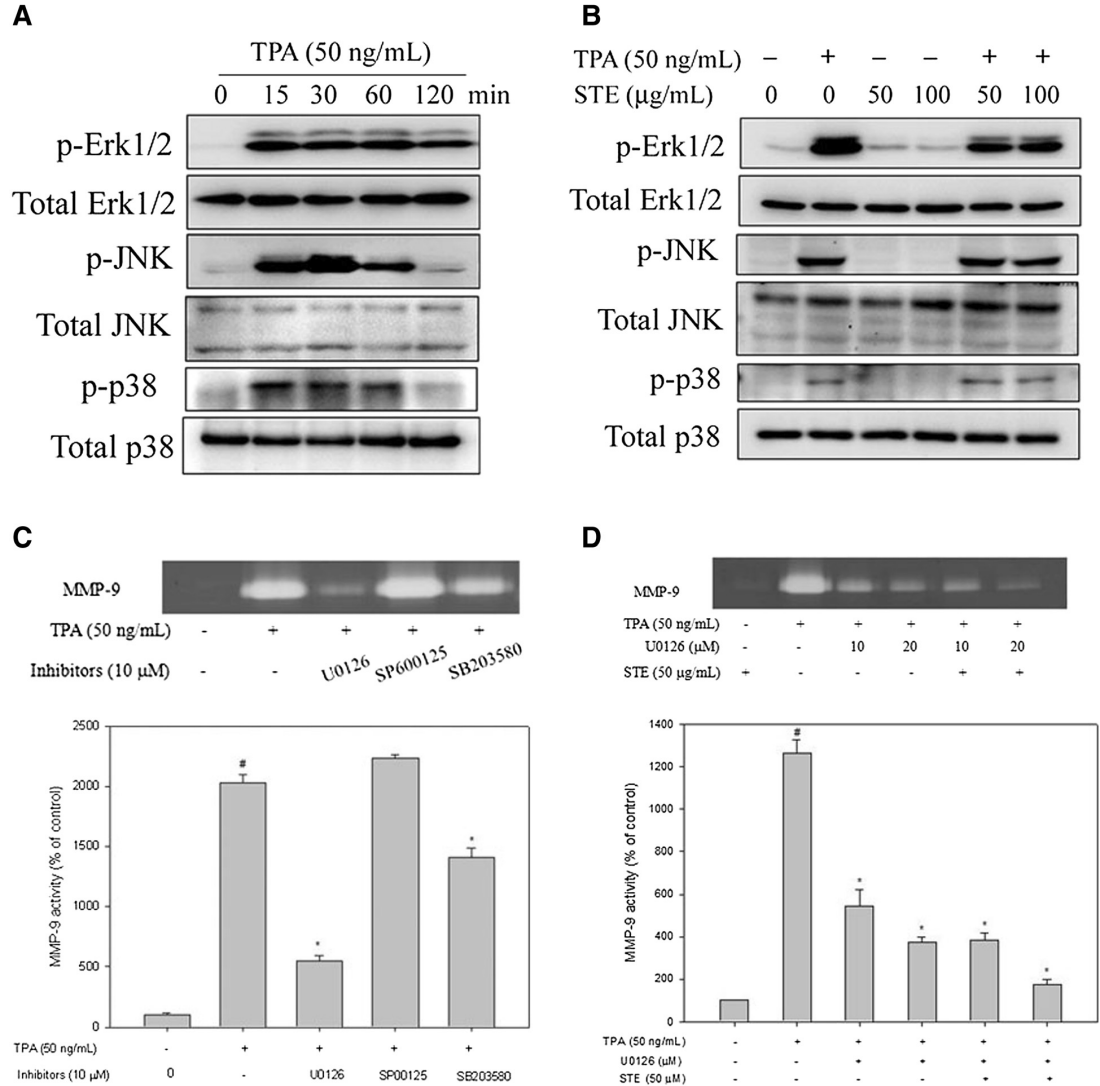
**Effects of TPA and STE on the MAPK pathway. (A)** HONE-1 cells were treated with TPA (50 ng/mL) for 15, 30, 60, and 120 min and then subjected to Western blotting to analyze the levels of Erk 1/2, JNK, and p38. **(B)** HONE-1 cells were treated with STE (0, 50, and 100 μg/mL) in the presence or absence of TPA (50 ng/mL) for 6 h and then subjected to Western blotting to analyze the levels of Erk 1/2, JNK and p38. **(C)** HONE-1 cells were pre-treated with U0126 (10 μM), SP600125 (10 μM) and SB203580 (10 μM) for 30 min and then incubated with TPA (50 ng/mL) for 24 h. The medium was subjected to gelatin zymography as described in the *Methods*. **(D)** HONE-1 cells were pre-treated with U0126 (10 or 20 μM) for 30 min and then incubated in the presence or absence of TPA (50 ng/mL) and STE (50 μg/mL) for 24 h. The medium was subjected to gelatin zymography. The values represented the means±SD of at least three independent experiments. *#p<*0.05 compared to the vehicle group; *p<0.05 compared to the TPA treatment group.

After the inhibitory effects of STE on cell migration/invasion and proteinases were revealed, the effects of STE on the expressions of MAPK pathways were investigated using Western blotting to elucidate their underlying mechanisms. Western blotting showed that TPA significantly increased the three MAPK pathway phosphorylations of HONE-1 cells in a time-dependent manner (*p*<0.05) (Figure 6A). Furthermore, STE reduced the phosphorylation of ErK1/2 in HONE-1 cells, but not the phosphorylation of the JNK and p38 pathways (Figures 6B).

To further determine whether STE inhibition of MMP-9 activity was caused mainly by the inhibition of the Erk 1/2 signaling pathway, its effects on a specific inhibitor of the Erk1/2 pathway (U0126) in HONE-1 cells were investigated. In the gelatin zymography assay, TPA-induced MMP-9 activity of HONE-1 cells was significantly reduced by the Erk 1/2 inhibitor (U0126) (Figure 6C). The results also showed that a combined treatment of the Erk 1/2 inhibitor and STE further reduced MMP-9 expression (Figure 6D). Thus, the inhibition of the Erk 1/2 signaling pathways might result in reduced MMP-9 expression.

## Discussion

The nasopharynx is situated over the base of the skull where lymphatic tissues and circulation are rich. Metastases of nasopharyngeal cancer cells to the neck lymph nodes and distant organs are common in NPC patients, even in the early stage of the disease. Herbal medicines are a popular practice of healthcare in eastern countries. Numerous studies have shown that they are beneficial in the treatment of many diseases, including cancers [23]. Although the anti-tumor activities of several herbal medicines against human NPC cells have been demonstrated previously [24,25], there is no data in current literature regarding the anti-metastatic activity of herbal medicine for NPC cells. The present study demonstrates that the extract of *Selaginella tamariscina* can significantly inhibit the migration and invasion ability of HONE-1 cancer cells, suggesting a potential role in the treatment of metastatic NPC.

A degradation of the ECM and components of the basement membrane by MMPs play a crucial role in the development of cancer metastasis. *In vivo* evidence from chicken chorio-allantoic membrane (CAM) assay shows that MMP-9 is inter-dependent in tumor invasion, while tumor cells show only low levels of invasion in the absence of MMP-9 [26]. Itoh et al. also report that metastatic colonies are seldom observed in MMP-9-deficient mice injected intravenously with melanoma or lung carcinoma cells [27]. Clinically, the positive relationships between MMP-9 and metastasis in patients with NPC have been reported [28,29]. The results in the present study demonstrate that STE inhibits the migration and invasion of human NPC HONE-1 cells via decreasing the MMP-9 protein levels (Figure 3). To date, this is the first scientific report related to the inhibitory effect of STE on NPC invasiveness via decreased production of tumor metastasis-related proteins. Since several studies have indicated that inhibition of MMP expressions or enzyme activities can be used as early targets for preventing cancer metastasis [30-32], *Selaginella tamariscina* may be a potential candidate for cancer treatment.

The expression of MMP-9 can be regulated by an inflammatory cytokine, a growth factor, or an oncogene through activation of different intracellular-signaling pathways [33]. Among these stimulators, TPA is a well-known substitute for diacylglycerol as a high affinity ligand for conventional protein kinase C (PKC). It can induce MMP-9 expression and result in an increase in invasion in various malignant cell lines [34]. FAK, one of the major kinases of focal adhesions, is a vital regulator involved in focal adhesion assembly and cell migration. FAK becomes activated when it is phosphorylated at tyrosine 397 (Y397). It is associates with Src and forms a dual kinase complex [35]. Activated Src phosphorylates FAK, thereby creating a signaling cascade through the Ras and mitogen-activated protein kinase (MAPK) [36].

Moreover, the activation of one or more MAPK pathways (e.g. ERK1/2, JNK and p38) is known to be important for the MMP-9 induction by TPA in various cell types [37-40]. The present study shows that the TPA-induced MMP-9 expression of HONE-1 cells is accompanied by an increase of phosphorylation of Src, FAK, ERK1/2, JNK and p38. The MAPKs inhibitors test suggests that TPA induces MMP-9 expression through the ERK1/2 and p38, but not the JNK, pathways. It can be assumed that the inhibitory effects of STE on TPA-induced MMP-9 activity of HONE-1 cells may be through the inactivation of the signaling pathways of TPA induction. The results here reveal that STE treatment of TPA-induced HONE-1 cells inhibit MMP-9 expression and ERK1/2 phosphorylation without affecting JNK and p38 phosphorylation (Figure 6). This indicates that participation of the Src/FAK/ErK 1/2 pathway is the putative mechanism for the inhibition of MMP-9 synthesis by STE in human NPC HONE-1 cells.

Several flavonoid compounds have been isolated from *Selaginella tamariscina*, including amentoflavone, apigenin, hinokiflavone, and sotetsuflavone [9,10,41]. Previous studies have shown that amentoflavone can reduce histamine release from rat peritoneal mast cells [42], while apigenin can inhibits the production of MMPs in various malignant tumors [43,44]. Oozes et al. report that pro-inflammatory signals are mediated by TNF-α via pathways of nuclear factor-κB (NF-κB) and Akt [45]. Ruiz and Haller have demonstrated that apigenin inhibits the pro-inflammatory gene expression in intestinal epithelial cells by blocking Akt phosphorylation/activity [46]. These findings suggest that flavonoids may be the active compounds responsible for the anti-metastatic activity of *Selaginella tamariscina*. Further investigations on the exact effective components of STE are warranted to determine its potential use in oncology.

## Conclusions

In conclusion, extract of *Selaginella tamariscina* prevents the metastasis of HONE-1 cells by the transcriptional inhibition of the MMP-9 expression and activity through a down-regulation of ERK1/2 signaling pathways. These results suggest that *Selaginella tamariscina* has the ability to exert inhibitory effects on critical steps in metastasis, including cellular mobility, migration and invasion. However, the interpretation of this study is limited because the lack of an *in vivo* animal study. *Selaginella tamariscina* should be further tested by an *in vivo* model to determine if it is effective in the prevention of nasopharyngeal carcinoma invasion or metastasis.

## Competing interests

The authors have no competing interests.

## Authors’ contributions

CHH, CWL, and MKC conceived and designed the study. BCW, SFY and CYC performed the experiments. YHH, HYH and HPL analyzed the data. CWL and MKC drafted the manuscript. CWL revised the manuscript. MKC provided comments and editorial review of the manuscript. All authors read and approved the final manuscript.

## Pre-publication history

The pre-publication history for this paper can be accessed here:

http://www.biomedcentral.com/1472-6882/13/234/prepub
